# Probing Multiple Transplant Delivery Routes of CD+34 Stem Cells for Promoting Behavioral and Histological Benefits in Experimental Ischemic Stroke

**DOI:** 10.1093/stcltm/szad081

**Published:** 2023-11-28

**Authors:** Jea-Young Lee, Justin Cho, Francesco D’Egidio, Christine Vignon, Hendrik Streefkerk, Matthieu de Kalbermatten, Ibon Garitaonandia, Cesar V Borlongan

**Affiliations:** USF Health Center of Excellence for Aging and Brain Repair, Tampa, FL, USA; USF Health Center of Excellence for Aging and Brain Repair, Tampa, FL, USA; USF Health Center of Excellence for Aging and Brain Repair, Tampa, FL, USA; CellProthera 12 Rue du Parc, 68100 Mulhouse, France; CellProthera 12 Rue du Parc, 68100 Mulhouse, France; CellProthera 12 Rue du Parc, 68100 Mulhouse, France; CellProthera 12 Rue du Parc, 68100 Mulhouse, France; USF Health Center of Excellence for Aging and Brain Repair, Tampa, FL, USA

**Keywords:** cerebral ischemia, cell transplantation, functional recovery, neurogenesis, angiogenesis, cell delivery route

## Abstract

Stroke is a leading cause of death in the US and around the world but with limited treatment options. Survivors often present with long-term cognitive and neurological deficits. Stem cell-based therapy has emerged as a potential treatment for stroke. While stem cell transplantation in stroke has reached clinical trials, mostly safety outcomes have been reported with efficacy readouts warranting more studies. In an effort to optimize the stem cell regimen for stroke, here we conducted vis-a-vis comparison of different routes of transplantation, namely, intracerebral, intraarterial, and intranasal delivery of expanded human CD34 + stem cells, called ProtheraCytes, in the established stroke model of transient middle cerebral artery occlusion (MCAO) using adult Sprague-Dawley rats. After adjusting for the dose and subacute timing of cell delivery, animals were randomly assigned to receive either ProtheraCytes or vehicle. Motor and neurological assays from days 7 to 28 post-stroke revealed significant functional recovery across all 3 delivery routes of ProtheraCytes compared to vehicle-treated stroke rats. Additionally, ProtheraCytes-transplanted stroke rats displayed significantly reduced infarct size and cell loss in the peri-infarct area coupled with enhanced neurogenesis and angiogenesis compared to vehicle-treated stroke rats. These results highlight the safety and efficacy of transplanting ProtheraCytes, including via the minimally invasive intranasal route, in conferring robust and stable behavioral and histological positive outcomes in experimental stroke.

## Graphical Abstract

**Graphical Abstract:**
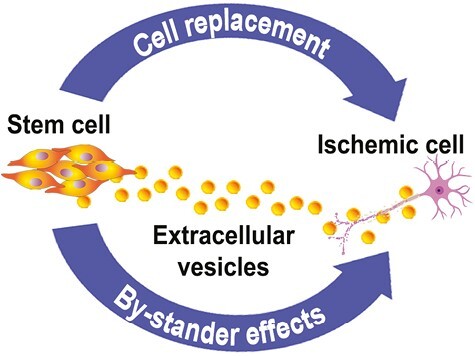
Stem cell-derived extracellular vesicles promote recovery of ischemic cells. The present study demonstrates a novel mechanism of action mediating stem cell-induced therapeutic effects in ischemic stroke. Whereas the long-standing view of stem cell repair of the stroke brain implicates cell replacement, our findings implicates a process of by-stander effects, whereby stem cells release extracellular vesicles (EVs), in particular CD-63-labeled EVs, which appear to target the vasculature and enhance vascular endothelial growth factor zlevels, while dampening inflammation (Iba-1 cells) and increasing neural cell proliferation (DCX). Altogether, these reparative processes rescue the neurovascular unit via a multi-pronged mechanism involving EV release, vasculogenesis, anti-inflammation, and neurogenesis.

**Significance Statement**

This study provides guidance on the safe and effective route of stem cell delivery in stroke animals, with mechanistic evidence suggesting the key role of extracellular vesicles in mediating the therapeutic effects of transplanted stem cells.

## Introduction

Stroke remains as one of the most detrimental diseases that cause significant neurological impairments.^1-3^ As the fifth leading cause of death in the US, stroke has limited treatment options, and the projected growth of vulnerable aging populations makes the search for effective stroke therapy even more urgent. Currently, endovascular thrombectomy and tissue plasminogen activator (tPA) are the only approved acute stroke treatment options. However, the short therapeutic time window and potential inducement of adverse effects limit the use of available acute stroke treatment.^1,4-11^ While rehabilitation offers some therapeutic effects, many chronic ischemic stroke victims are unable to fully recover cognitive and motor functions due to loss of brain cells caused by the deprivation of oxygenated blood.^12,13^ With 87% of all stroke cases consisting of ischemia^12^ and current stroke treatments offering limited use, a potent treatment that can regenerate lost brain cells is warranted.

Stem cell therapy has emerged as a potential candidate to treat stroke by restoring lost brain cells. The neuroprotective and regenerative abilities of stem cells in both acute^14-17^ and chronic^18-20^ events have marked stem cell transplantation as a novel solution to stroke-induced deficits. Neurotrophic factor secretion, restorative properties, cell replacement, and biobridge formation are some of the functional mechanisms of stem cell therapy.^20-29^ In middle cerebral artery occlusion (MCAO) models in rats, different types of cells, such as bone marrow and umbilical cord blood-derived mesenchymal stem cells (MSCs),^30-32^ demonstrate similar therapeutic effects by increasing neurotrophic growth factors, decreasing apoptosis and reducing neurological damage in infarct border zone.^16,30,33-35^ Bone marrow stromal cells also increase vascular endothelial growth factor (VEGF) activity and enhance angiogenesis.^32^ Umbilical cord blood cells have shown functional recovery in MCAO models, but there are limited clinical studies to support their use in humans.^31^ Similarly, MSCs have been advantageous for their accessibility, but their performance is highly dependent on their method of transplantation,^30^ and their safety and efficacy remain undetermined.^36^ Significant improvements to angiogenesis, neurogenesis, and vasculogenesis accompany MCAO mice treated with endothelial cells.^14^ While stem cell transplantation possesses potent mechanisms outlined in MCAO models, finding the most suitable line of stem cells proves to be a challenge.

As previously shown in acute myocardial infarction, acute cerebral ischemic attacks are followed by large and bursting mobilizations of peripheral blood derived CD34 + cells at 1-3 days and 7-10 days after the event.^37^ The extent of the CD34 + cell mobilization is significantly correlated to the neurological and functional recoveries observed at one and 3 months in the NIH Stroke Scale (NIHSS) and modified Rankin Scale (mRS), respectively, therefore being predictive of neurological and functional recovery.^37^ Indeed, a clinical study that mobilized CD34 + cells via daily subcutaneous injections of granulocyte colony stimulating factor (G-CSF) for 5 consecutive days after ischemic stroke shows functional and structural improvement in some patients.^38^ When 1-3 × 10^6^ CD34 + cells were injected intraarterially by catheter angiography into the ipsilesional middle cerebral artery within 7 days of stroke onset, all patients displayed improvements in mRS and NIHSS score at 6 months.^39^ Additionally, when 3-8 × 10^6^ CD34 + cells were injected intracerebrally at ≥ 6 months after stroke onset in patients with a middle cerebral artery infarct, treated patients exhibit significantly greater improvement in NIHSS, mRS, and European Stroke Scale at 12 months post-treatment compared to control patients.^40^ CD34 + cells have been shown to promote angiogenesis via the secretion of paracrine factors such as exosomes containing pro-angiogenic miRNAs^41^ and via gap junction mediated cell-cell interaction.^42^ Preclinical studies have shown that administration of CD34 + cells after stroke enhances neurogenesis via angiogenesis.^43^ CD34 + cells promote an environment conducive to neovascularization of the ischemic brain that enhances neuroplasticity and neuronal regeneration.^44^

While the success of stem cell therapy heavily relies on the type of stem cell used, the method of transplantation is equally, if not more, important due to different delivery routes triggering different therapeutic mechanisms and presenting unique functional benefits. Intracerebral (IC) transplantation is known for its high number of stem cells in the lesion area, significant neurological recovery, and lower peripheral side effects.^45,46^ However, the downside of IC delivery is its limited clinical application for stroke patients who cannot tolerate direct injection via neurosurgical operations, which may lead to surgical complications.^47^ Intra-arterial (IA) injection offers a less invasive method of delivery for more vulnerable stroke patients. Due to stem cells’ homing capability, indirect injection of stem cells can still penetrate the blood-brain barrier and reach the lesion site.^48^ IA infusions in phase I/II studies improved functional outcomes over 12 months.^49^ Intravenous (IV) administration also achieves similar therapeutic outcomes as IA by avoiding surgical interventions. However, both IA and IV methods have lower effectiveness, compared to IC delivery, as fewer cells arrive at the infarct area. Additionally, IA and IV injections increase the risk of pulmonary embolism and thrombosis as a large number of stem cells accumulate in the lungs and spleen.^48,50,51^ IA administration of some cells, in particular MSCs, has the risk of secondary cerebral infarcts/ microembolism.^52^ Intranasal (IN) route has emerged as a new method of stem cell transplantation. Because stem cells are administered through the olfactory system, cells can bypass the blood-brain barrier and reach the lesion site.^53,54^ IN administration in ischemic injury models has demonstrated improved cognitive, motor, and sensory functions through noninvasive operations,^55^ highlighting its safety and convenience without sacrificing its effectiveness. Further research is needed to solidify IN as a novel delivery route for stem cell therapy.

Extracellular vesicles (EV) play a notable role in intercellular communications, such as coagulation and immune responses.^56^ Cells undergoing apoptosis tend to release vesicles to the extracellular environment, but recent discoveries suggest that healthy cells also release vesicles to the extracellular environment. Aging animals treated with small EVs (sEVs) derived from adipose mesenchymal stem cells (ADSCs) demonstrated improvement in age-dependent functions.^57^ ADSC-sEVs also promoted regenerative effects in the kidneys and muscles by lowering inflammatory activity, oxidative stress, and senescence markers.^57^ Furthermore, tissues of aged mice treated with ADSC-sEVs had lower predicted epigenetic age and metabolome similar to younger mice.^57^ MicroRNAs in sEVs may be responsible for the therapeutic effects.^57^

EVs can be classified based on their cellular origin: exosomes, microvesicles, and apoptotic bodies.^56,58^ Healthy, functional cells release both exosomes and microvesicles. Exosomes are endocytic vesicles that form through inward budding of multivesicular endosomes while microvesicles bud from the cell surface. Exosomes are favored over microvesicles in identifying healthy cell activity due to their consistent size and known protein content.^59^ Tetraspanins, such as CD63, are a branch of membrane proteins that congregate into the microdomains of the plasma membrane.^58,60,61^ They can be used as markers for EV due to their abundance in exosomes, signifying healthy cell activity.

This study was designed to investigate the different routes of transplantation, namely IC, IA, and IN delivery of expanded human CD34 + stem cells ProtheraCytes .^62^ Here, we assess the safety and efficacy of ProtheraCytes transplantation, including via the minimally invasive IN route, in behavioral and histological outcomes in the MCAO stroke model.

## Methods

### Animals

**Figure 1.**
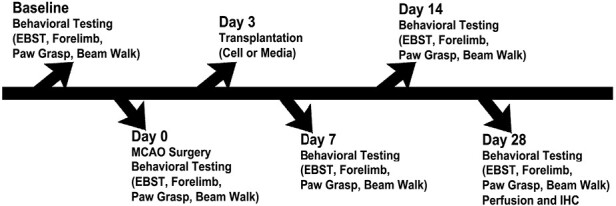
A timeline of the experimental procedures. Stem cell transplantation was performed on day 3. Behavior testing was performed on day −1 (baseline), 0, 7, 14, and 28. Middle cerebral arterial occlusion (MCAO) was performed on day 0.

All experiments were conducted in accordance with the National Institutes of Health Guide and Use of Laboratory Animals. The experiments were approved by Institutional Animal Care and Use committee (IACUC approval number: IS00009075) of the University of South Florida, Morsani College of Medicine. All animals had free access to food and water while housed under normal conditions (20 °C, 50% relative humidity, and a 12 hours light/dark cycle). A total of 60 adult Sprague-Dawley rats (250 g male, approximately 8 weeks old) equally divided in 6 groups were used (Figure 1). Of all the animals, 3 rats died at day 7, 2 of which without undergoing any transplantation.

### MCAO model

Adult Sprague-Dawley rats were subjected to stroke (*n* = 60) and anesthetized by a mixture of 1%–2% isoflurane in nitrous oxide/oxygen (69%/30%) via face mask. Body temperatures were maintained at 37 ± 0.3 °C during the surgical procedures. A midline skin incision was made in the neck with subsequent isolation of the left common carotid artery, the external carotid artery (ECA), and internal carotid artery. Thereafter, a 4-0 monofilament nylon suture (4-0 Medium B MCAO suture, Doccol Co.) was advanced from the common carotid artery bifurcation until it blocked the origin of the MCA. The skin incision was closed with surgical clips. Animals were allowed to recover from anesthesia during the 1-hour MCAO. At 1 hour after MCAO, animals were re anesthetized, and reperfusion commenced with the withdrawal of the suture. Thereafter, surgical incisions were closed, and animals were allowed to recover from anesthesia. Regarding pain management, animals underwent a single injection of Meloxicam ER (2 mg/mL) before surgery and a single injection of Buprenorphine SR (1.2 mg/kg) after surgery.

### CD34 + Cell Culture for Collection of ProtheraCytes

ProtheraCytes were obtained after expansion of mobilized CD34^+^ cells from healthy donors (Lonza) as previously described.^63^ The transport of ProtheraCytes from CellProthera (Mulhouse) to the USF Health Center was conducted at +4/10 °C with temperature monitoring.

### Transplantation of ProtheraCytes

At 3 days after MCAO, animals were randomly assigned to receive transplantation of ProtheraCytes at either IA (3 million cells in 1 mL vehicle; *n* = 10), IC (300k cells in 10 μL vehicle; *n* = 10), or IN (1 million cells in 36 μL vehicle; *n* = 10) or vehicle alone (saline intraarterially, intracerebrally, or intranasally; *n* = 30). IA was via the carotid artery. Since the animals received right MCAO, animals also received infusion of cells/saline into the carotid artery. Animals were anesthetized with a mixture of 1%-2% isoflurane in nitric oxide/oxygen (69%/30%) via a face mask, and body temperature was maintained at 37 ± 0.3 °C during the surgical procedures. Infusion of cells/saline was performed via a bolus injection delivered from a 25G needle of a 1 mL syringe inserted into the carotid artery. The syringe was filled with cells/saline. After dosing, the needle was removed and the carotid artery pressed for 30 seconds to prevent any bleeding, and the skin wound reclosed with surgical clips. For IC transplantation, stereotaxic surgery targeted the right striatum. All surgical procedures were conducted under aseptic conditions. Animals were anesthetized with a mixture of 1%-2% isoflurane in nitric oxide/oxygen (69%/30%) via a face mask, and body temperature was maintained at 37 ± 0.3 °C during the surgical procedures. Once deep anesthesia was achieved (by checking for pain reflexes), hair was shaved around the area of surgical incision (skull area) with enough border to prevent contaminating the operative site, followed by 2 surgical germicidal scrubs of site, and draping with sterile drapes. The animal was then fixed to a stereotaxic apparatus (Kopf Instruments). A 26-gauge Hamilton syringe was then lowered into a small, burred skull opening (transplant coordinates were adjusted to correspond with the striatal area adjacent to the infarcted site: 0.5 mm anterior and 1.0 mm lateral to bregma and 4.0 mm, 3.5 mm, and 3.0 mm below the dural surface). Within this single needle pass, 3 deposits of the test article (100 000 cells in 3 μL per deposit or a total of 300 000 cells in 9 μL of saline for 3 deposits) were made. The target area was the medial striatum which corresponds to the peri-infarcted striatal area, based on previously established target sites for similar stereotaxic implants. Each deposit consisted of 100,000 viable cells in 3 μL volume infused over a period of 3 minutes. Following an additional 2-minute absorption time, the needle was retracted, and the wound closed with stainless steel wound clips. A heating pad and a rectal thermometer allowed maintenance of body temperature at approximately 37 °C throughout surgery and following recovery from anesthesia. For IN transplantation, animas were held with a hand grip that allowed the animals to recline on their backs while immobilizing the skull, and the nose drop containing the substance/cell suspension was carefully placed on one nostril allowing it to be snorted naturally, and then the other nostril. One hundred units of hyaluronidase (Sigma-Aldrich Chemie GmbH, H3506) dissolved in 24 μL sterile PBS was administered to the rat nostrils (6 μL/nostril, repeat once after 2 minutes) 30 minutes prior to the administration of ProtheraCytes or vehicle. One million cells resuspended in 36 μL were applied to each rat using the same method as described for hyaluronidase, while control group received the same amount of vehicle only. Immunosuppressants were not required after cell transplantation for MSCs therapeutic effects, as already demonstrated.^14,24^

### Behavioral Tests

All investigators were blinded to the treatment conditions when testing the animals. Each rat was subjected to a series of behavioral tests to assess the motor and neurological conditions of the animals before (baseline) and after MCAO (day 0) and after perfusion (days 7, 14, and 28). Behavioral tests included the elevated body swing test (EBST), forelimb akinesia test, bilateral forepaw grasp, and beam walking ability tests.

#### Elevated Body Swing Test (EBST)

EBST is a measure of asymmetrical motor behavior that does not require animal training or drug injection. The rat was held in the vertical axis approximately 1 inch from the base of its tail and then elevated to an inch above the surface on which it had been resting. The frequency and direction of the swing behavior were recorded over 20 tail elevations. A swing was counted when the head of the rat moved more than 10° from the vertical axis to either side. Normally, intact rats displayed a 50% swing bias, that is, the same number of swings to the left and to the right. A 75% swing bias toward one direction was used as a criterion of motor deficit. The total number of swings made to the biased side was added per animal and divided by 20, providing the average number of biased swings per individual animal.

#### Forelimb Akinesia

Forelimb akinesia measures the ability of the animal to hold on to a 2 mm diameter steel rod to assess the ipsilateral and contralateral forepaw strength and motility. Grades were as follows: 0 for normal grasping behavior, 1 for slow grasping without rigidity, 2 for slow grasping with rigidity, and 3 for no grasping with forelimbs.

#### Bilateral Forepaw Grasp

Bilateral forepaw grasp measures the ability of a rat to hold onto a 2 mm diameter steel rod. Grade 0 was used for rats with normal forepaw grasping behavior, 1 for rapid grasping but with rigidity, 2 for slow grasping with rigidity, and 3 for a rat unable to grasp with the forepaw.

#### Beam Walking

Beam walking ability uses a beam apparatus that is 80 cm long with a flat surface of 2-4 cm width resting at least 40 cm above the table/surface top on 2 poles. The animal was placed at one end of the beam then the ability to traverse the beam and reach the other end was assessed. The grades were as follows: 0 for a rat that easily traverses the beam, 1 if the rat slowly traverses the beam, 2 for partially traversing the beam but falls off, and 3 for a rat unable to stay on the beam for 10 seconds.

The scores from all 3 tests were added to give a total neurologic deficit score (maximum possible score is 9 with mean composite neurologic score of 3). A score of 2.5 was set as a criterion to be considered a “stroke” animal.

### Euthanasia and Perfusion

To perform immunofluorescent analysis, animals were euthanized under deep anesthesia. Animals were briefly perfused through the ascending aorta with cold PBS (200 mL), followed by 4% paraformaldehyde in phosphate buffer (200 mL). Rat brains were harvested and postfixed in the same fixative (72 hours), followed by complete submersion in 30% sucrose in phosphate buffer. Cryostats were used to cut multiple coronal sections at 40 μm thickness, which were stored at −20 °C.

### Nissl Staining

Nissl staining was performed with 0.1% cresyl violet solution (Sigma-Aldrich) using a standard protocol to evaluate the peri-infarct injury of our MCAO model. From each perfused brain, 6 coronal sections between the anterior edge and posterior edge of the MCAO infarct area were collected and processed for Nissl staining. Every 6th coronal tissue section was chosen at random to quantify cell survival in the peri-infarct area. Brain sections were examined using a light microscope (Keyence). Neuronal survival in the peri-infarct area of the brain was quantified using a computer-assisted image analysis system (NIH Image Software) and was expressed as a percentage of the ipsilateral hemisphere compared to the contralateral hemisphere. The infarction area ratio calculated defining left hemisphere (LT), right hemisphere (RT), and infarction area (RI) in mm^2^. Infarction area ratio = [LT − (RT − RI)] × 100/LT (%).^64^ To control for edema, the following edema correction formula was performed in this study. The volume of brain damage was measured in each slice and quantified by a computer assisted image analysis system (NIH Image Software) and calculated by the following formula: 2.0 mm (thickness of the slice) × (sum of the damaged area per slice or in all brain slices) then expressed as percentage. To minimize artifacts produced by post-TBI edema in the injured area, the brain damage volume was calculated as described previously.^65^ Briefly, the injured area in the ipsilateral hemisphere was indirectly measured by subtracting the non-injured area in the ipsilateral hemisphere from the total intact area of the contralateral hemisphere. We counted positive live cells based on their morphological features, in particular taking into consideration their round and oval shape soma with a healthy nucleus as opposed to those cells with dense, compact, or fragmented nucleus representative of a pyknotic cell.

### Immunohistochemistry

Staining for vascular endothelial growth factor receptor 1 (VEGFr1) (1:200; Abcam; AB32152), ionized calcium binding adaptor molecule-1 (IBA-1) (1:500; Wako; 019-19741), doublecortin (DCX) (1:500; Abcam; AB18723), CD63 (1:200; Novus Biologicals; NB100-77913) were conducted on every 1 of 6 sections, 40 mm in thickness of brain. From each section, approximately 4-6 images at 20× magnification were taken using a confocal microscope (Zeiss) and analyzed with ImageJ (National Institutes of Health). All photomicrographs were converted to gray scale. Background was selected from blank control images and subsequently used to subtract the background from all images. The same threshold was used for all images. Thereafter, the labeling intensity of each section was quantified as the average optical density readings of 4-6 randomly selected areas within that section. The final labeling intensity of each group was expressed as the average of each labeling intensity per section. All sections were washed in PBS for 5 minutes 3 separate times. After washing, samples were then blocked for 60 minutes at room temperature with 5% normal goat serum (Invitrogen) and 0.1% triton X-100 in 0.1 M PBS. Endothelial progenitor cell marker with transplantation cell marker mouse monoclonal human CD34 (1:200; Thermo Fisher Scientific, MA5-15331), which specifically cross-reacted only with human samples without rat cross-reactivity, were double-labeled to assess ProtheraCytes. Then, brain slices were incubated overnight with the listed primary antibodies at 4 ˚C. Afterward, sections were washed 3 times for 10 minutes in 0.1 M PBS and soaked in 5% normal goat serum and 0.1% triton X-100 in 0.1 M PBS with corresponding secondary antibodies, goat anti-mouse IgG-Alexa 488 (green) (1:500; Invitrogen), or goat anti-rabbit IgG-Alexa 594 (red) (1:500; Invitrogen) for 2 hours. Sections were again washed 3 times for 5 minutes in PBS, and cover-slipped with Vectashield hardset with DAPI (H-1500, Vector Laboratories, California). All sections were examined on the ipsilateral corpus callosum using a confocal microscope (Zeiss) and cell counting was done by a blinded team member. Control studies included exclusion of primary antibody substituted with 5% normal goat serum in 0.1 M PBS.

### Exosome Isolation From ProtheraCytes

ProtheraCytes were seeded in a serum-free medium (DMEM medium) in a bioreactor for extracellular vesicle release by exerting a controlled mechanical stimulation on cells for 30 minutes to 2 hours according to Everzom’s proprietary method. The size distribution and concentration measurements of ProtheraCytes-derived exosomes were performed using the Nanosight (NS300). The exosome membrane markers were quantified by flow cytometry using the MACSPlex Exosome kit (Miltenyi).

For miRNA analysis, ProtheraCytes were cultured at the concentration of 2.5 × 105 cells/mL in StemSpan-AOF (STEMCELL Technologies) supplemented with cytokines for 40 hours. Then, cells were collected by centrifugation at 400*g* for 10 minutes; and exosomes were purified from the supernatant by precipitation using the ExoQuick-TC kit (System Biosciences) according to the manufacturer’s instructions. After isolation, exosomes were characterized by flow cytometry using the ExoStep kit with a bead-bound anti-CD63 capture (ImmunoStep, Spain) confirming the identity of the exosomes secreted by ProtheraCytes.

### Oxygen Glucose Deprivation (OGD)

Once confluence of primary rat cortical cells was achieved, we initiated the OGD. The cells were initially exposed to Dulbecco’s phosphate-buffered saline, then placed in an anaerobic chamber (Plas-Labs, Inc) containing nitrogen (95%) and carbon dioxide (5%) for 15 minutes at 37 °C, and finally, the chamber was sealed and incubated for 90 minutes at 37 °C (hypoxic–ischemic condition). OGD was terminated by changing normal media, and cell cultures reintroduced to the regular CO_2_ incubator (normoxic condition) at 37 °C for 1 h, which represented a model of reperfusion. After reperfusion, wells were randomly cocultured with ProtheraCytes or standard DMEM medium, at a concentration of 40 000 cells per well overnight. This coculture set-up, the primary rat cortical cells were suspended in the treatment condition using 8-well poly-L-lysine plates, with each treatment condition done in 6 biological samples. The coculture was created using a 2-chamber system with the primary rat cortical cells in lower chamber and the ProtheraCytes in the upper chamber, allowing us to conduct accurate assessments of the primary rat cortical cells without contamination from the ProtheraCytes population. Cell viability (Trypan blue) and metabolic activity (3-[4,5-dimethylthiazol-2-yl]-2,5 diphenyl tetrazolium bromide or MTT) levels were examined in neurons.

#### Trypan Blue Assay

Trypan blue (0.2%) exclusion method was conducted and mean viable cell counts were calculated in 4 randomly selected areas (1 mm^2^, *n* = 10) to reveal the cell viability after the ischemic-reperfusion condition. Briefly, within 5 minutes after adding trypan blue, we digitally captured under microscope (×200) 10 pictures (approximately 100 cells per picture) for each condition, then randomly selected 5 pictures, and counted the number of cells for each individual treatment condition. Normalized cell viability was calculated from the following equation: viable cells (%) = [1.00 − (number of blue cells/number of total cells)] × 100.

#### MTT-cell Viability Assay

The colorimetric 3-(4,5-dimethylthiazol-2-yl)-2, 5-diphenyltetrazolium bromide or MTT reduction assay was conducted by following the instructions for use of Promega Corporation products (Cell Titer 96, Non-Radioactive Cell Proliferation Assay, Promega Corporation). This method assessed mitochondrial activity, thus an indirect measure of cell viability by measuring the ability of cultured cells to convert yellow MTT to purple formazan dye. The supernatant and the cells were separated from the mixed culture at the end of the 3-hours exposure time. Approximately 100 μL DMEM without phenol red was added, then 20 μL of the dye solution was added to each well, and the mixture was incubated on the plate at 37 °C for 3 hours in a humidified, 5% CO_2_ atmosphere. After incubation, 100 μL of the solubilization solution/stop mix was added to each well, and the plate was allowed to stand overnight in the humidified, 5% CO_2_ incubator at 37 °C. The absorbance was quantified spectrophotometrically at a wavelength of 570 nm and with a reference wavelength of 900 nm in the BioTek Synergy HT 96-well microplate reader (BioTek Instruments, Inc.).

### Statistical Analysis

The statistical data was obtained using one-way analysis of variance (ANOVA) and subsequent post hoc Bonferroni’s test for behavior tests. Statistical significance was preset at *P* < .05. (GraphPad version 9.0) (means ± S.D.). The Kolmogorov-Smirnov test was used to assess normality and the resulting values were <5% of the critical values.^66^

## Results

### ProtheraCytes ameliorate stroke-induce behavioral deficits

**Figure 2.**
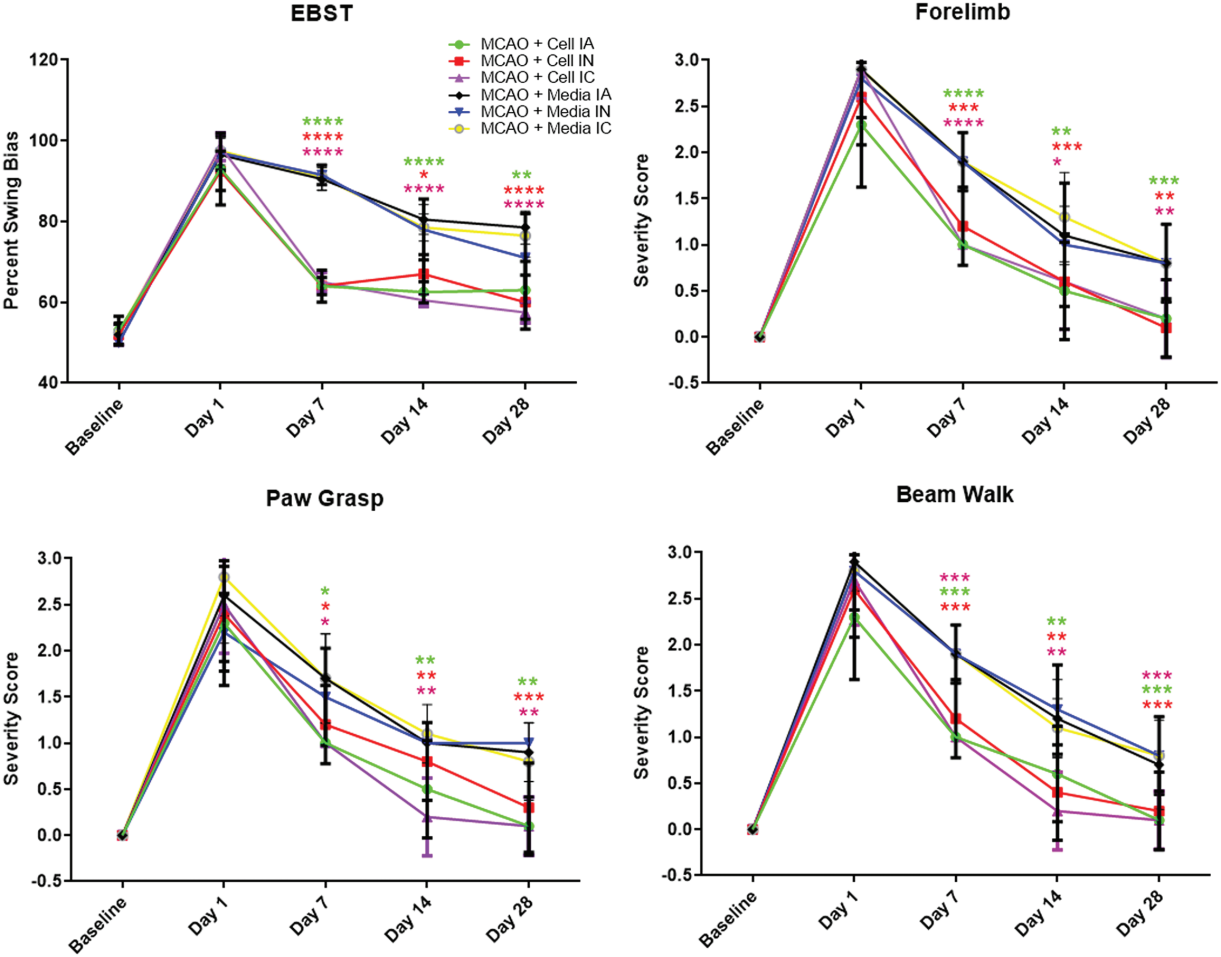
Behavioral tests. (a) Motor activity revealed by EBST. MCAO + cell groups displayed significantly less asymmetry on days 7, 14, and 28 (*P < .05, **P < .01, ****P < .0001). (b) Motor activity revealed by cylinder test. MCAO + cell groups demonstrated significantly more use of impaired forelimb (*P < .05, **P < .01, ***P < .001, ****P < .0001). (c) Motor activity revealed by Grip Strength. MCAO + cell groups presented significantly less impaired paw grasp (*P < .05, **P < .01, ***P < .001). (d) Motor activity revealed by balance beam. MCAO + cell showed significantly better motor coordination during beam walks (**P < .01, ***P < .001).

A combination of regular motor and neurological tests vulnerable to stroke was conducted to examine the post-stroke functions and stem cell treatment. All animals displayed a strong swing bias in EBST and impaired scores in balance beam, paw grasp, and forelimb akinesia tests on day 0 after MCAO. Neurological and motor tests were also performed on days 7, 14, and 28 after transplanting treatment groups with ProtheraCytes on day 3. Post-transplantation results revealed that animals treated with ProtheraCytes displayed significantly improved functional outcomes in all motor tests examined here (Fig. 2). EBST was performed to evaluate locomotor performance after MCAO and cell transplantation. Stroke animal groups treated with ProtheraCytes had significantly lower percent swing bias (*F*_20,270_ = 19.69, ****P < .0001) compared to the vehicle-treated animal groups. No significant difference was found among IC, IA, and IN transplanted groups. The beam walking test showed that ProtheraCytes-treated stroke groups showed significantly prolonged time spent balancing on the rod (*F*_20,270_ = 3.568, ****P < .0001) compared to vehicle-treated stroke animals while no significant difference among IC, IA, and IN transplanted cell groups was noted. Paw grasp test revealed significantly lower stroke severity scores in ProtheraCytes-treated stroke animals (*F*_20,270_ = 3.480, ****P < .0001) compared to vehicle-treated stroke animals even after 28 days. There was no significant difference among the IC, IA, and IN transplanted groups in paw grasp tests. Similarly, the forelimb test revealed lower stroke severity scores in cell transplanted stroke animal groups (*F*_20,270_ = 2.996, ****P < .0001) compared to vehicle-treated groups. Again, no significant differences were found among IC, IA, and IN treatment groups. These results highlight the significant improvement in motor and neurological performances in stroke animals treated with ProtheraCytes compared to vehicle-treated stroke animals without significant differences in the delivery routes of the stem cells.

### ProtheraCytes Reduce Cerebral Infarcts and Peri-Infarct Cell Loss

**Figure 3.**
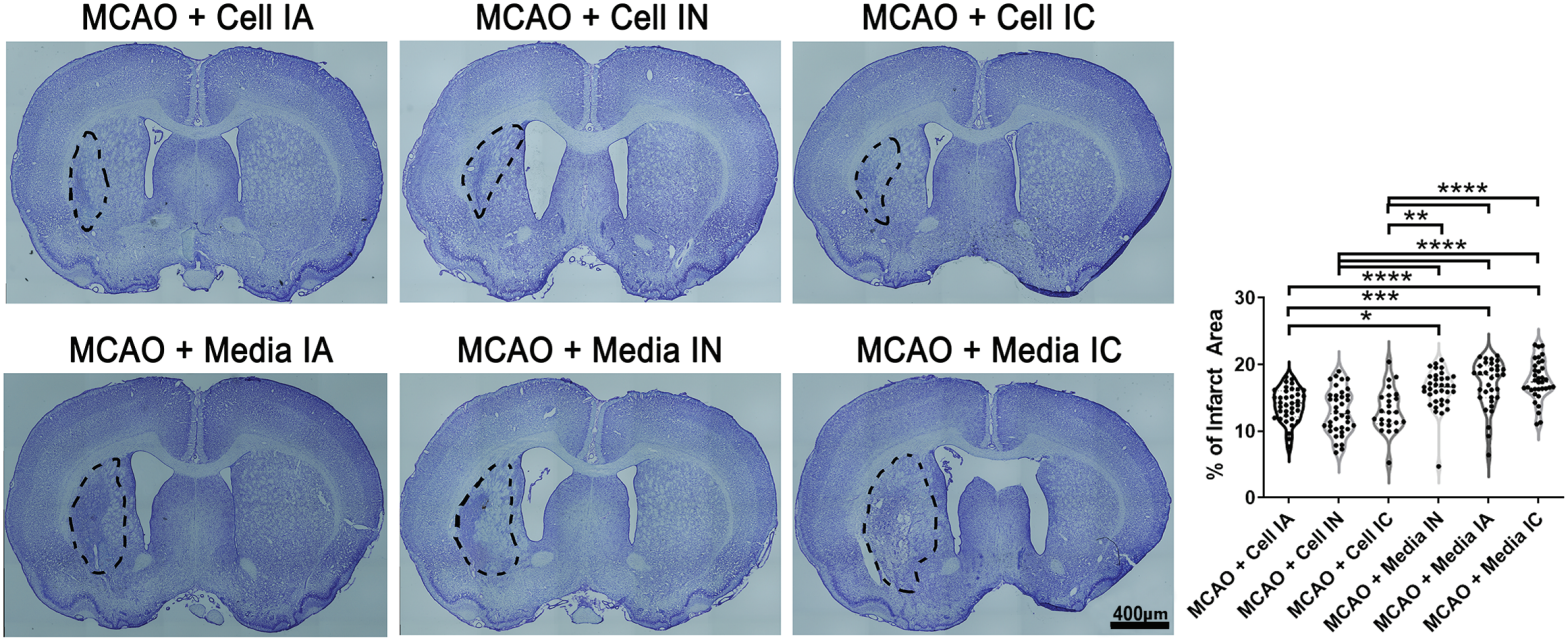
Infarct area. Nissl staining for coronal brain sections showing infarct areas (black outline) of MCAO + Cell IA, MCAO + Cell IN, MCAO + Cell IC, MCAO + Media IA, MCAO + Media IN, MCAO + Media IC. MCAO + Cell groups displayed significantly smaller infarcts (*P < .05, **P < .01, ***P < .001, ****P < .0001).

**Figure 4.**
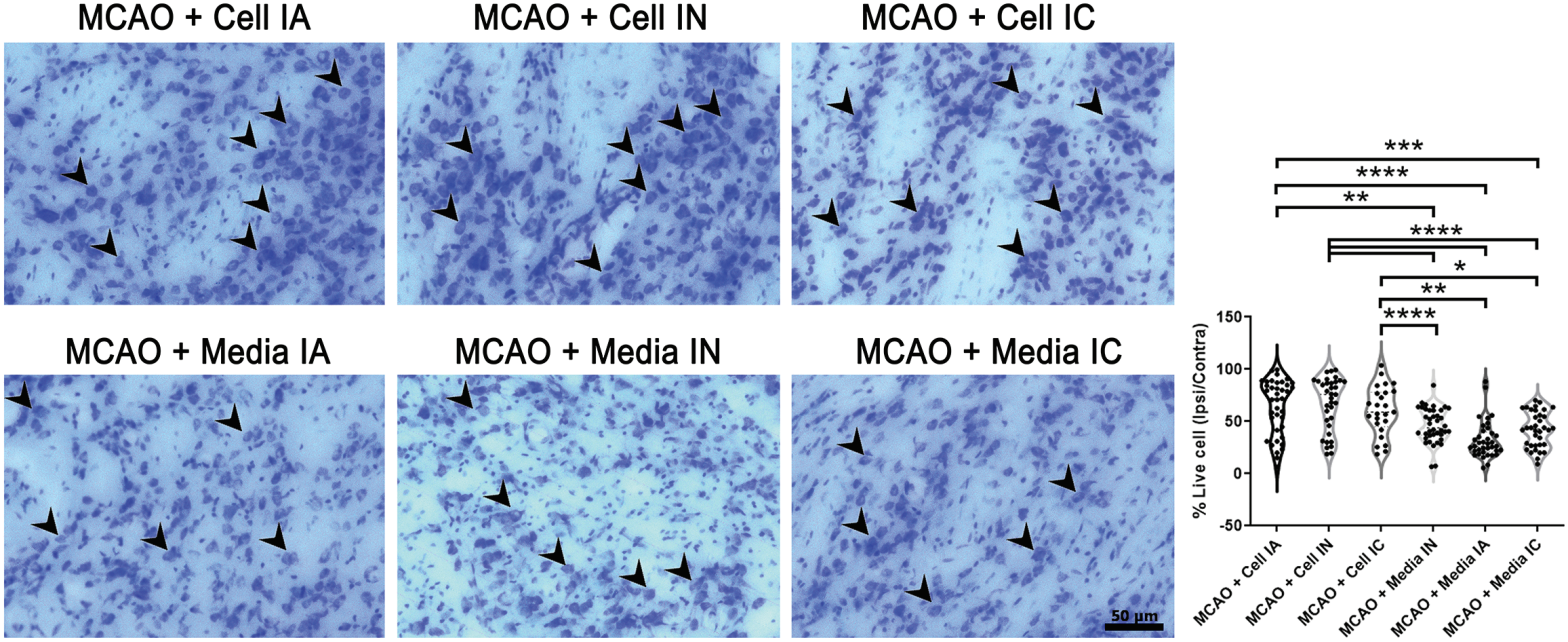
Peri-infarct area. Nissl staining for quantitative analysis of live cells (black arrow heads) in the peri-infarct area for MCAO + Cell IA, MCAO + Cell IN, MCAO + Cell IC, MCAO + Media IA, MCAO + Media IN, MCAO + Media IC. MCAO + Cell groups showed significantly more living cells in the peri-infarct (*P < .05, **P < .01, ****P < .0001). Scale bar = 50 µm.

Measurement of the infarct area and evaluation of neuronal survival in the peri-infarct area of the cortex was performed through Nissl staining with 0.1% cresyl violet to stain neuronal Nissl bodies. Compared to the control group, the stroke animals transplanted with ProtheraCytes presented with a reduced cerebral infarct area (*F*_5,196_ = 14.64, *P < .05, **P < .01, ***P < .001, ****P < .0001; Fig. 3). No significant difference in the infarct area was found among IC, IA, and IN transplanted groups. Additionally, ProtheraCytes-treated groups displayed significantly higher percentage of live cells in the peri-infarct cortex (*F*_5,206_ = 15.14, *P < .05, **P < .01, ***P < .001, ****P < .0001) compared to vehicle-treated stroke animals (Fig. 4). There was no significant difference in the peri-infarct area live cell percentage among IC, IA, and IN delivered cell transplantation. Observations made in the infarct and peri-infarct areas indicate a significant reduction in neuronal damage in ProtheraCytes-treated stroke animals compared to vehicle-treated stroke animals without difference in the method of transplantation.

### ProtheraCytes Dampen Stroke-Induced Inflammatory Response in the Brain

**Figure 5.**
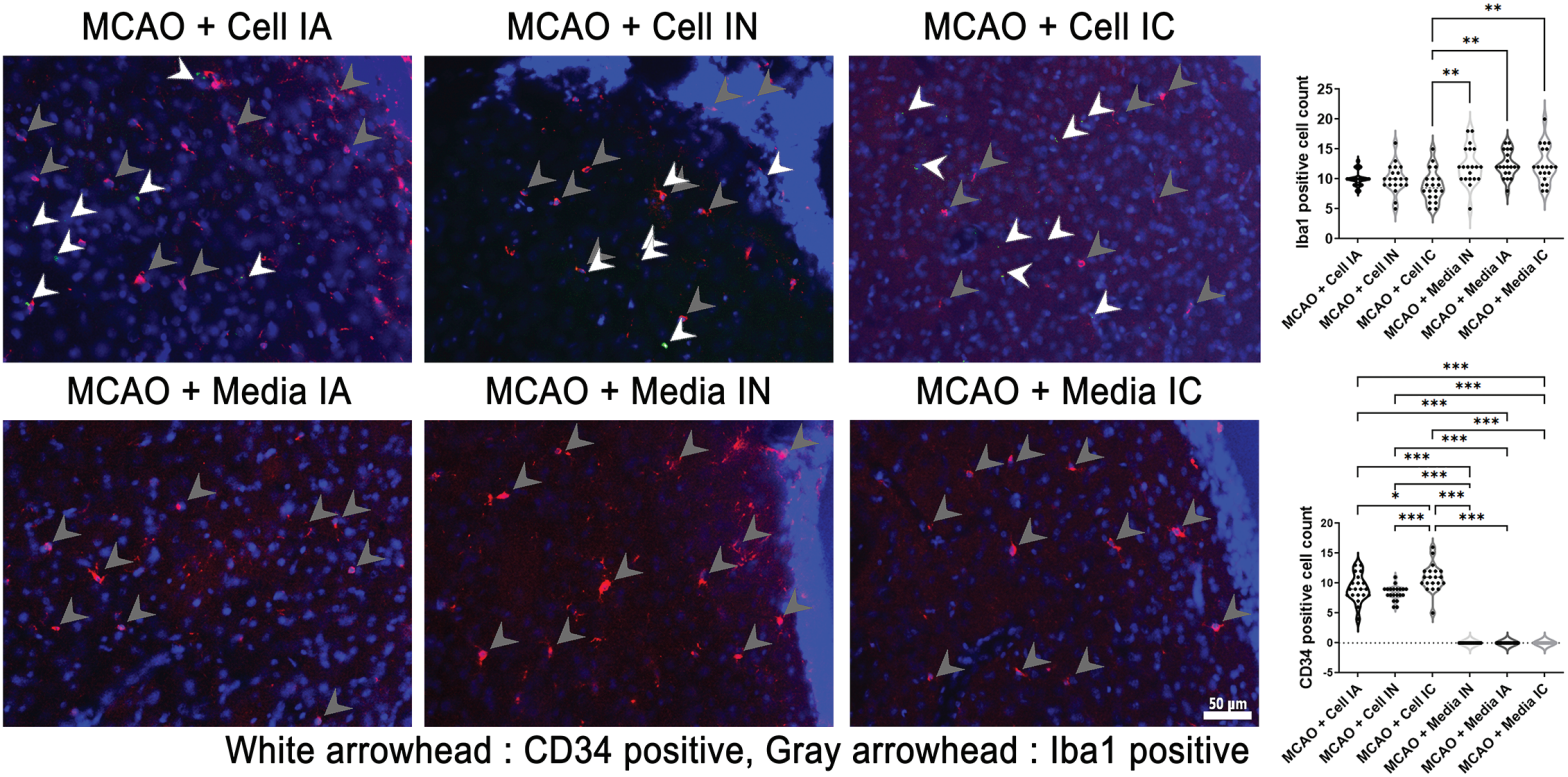
Inflammation. Iba-1 double stained with CD34 to measure inflammatory activity. MCAO + Cell IC had significantly less Iba-1 positive cells than MCAO + media IN, MCAO + media IA, and MCAO + media IC groups (**P < 0.01). Treatment groups had significantly more CD34 expression (***P < .001) with MCAO + Cell IC having the highest cell count within the treatment group (*P < .05; ***P < .001). Scale bar = 50 µm.

Iba-1 with endothelial progenitor cell marker with transplantation cell marker (CD34) staining was used to quantify neuroinflammation and microglial cell activity in the peri-infarct area (Fig. 5). Stroke animals treated with IC transplanted ProtheraCytes displayed a significant reduction in Iba-1 positive activated microglial cells in the peri-infarct cortex (*F*_5,114_ = 7.130, ***P < .001). Reduced Iba-1 positive cells were also seen in treatment groups via IA and IN administration, no significance was found when compared to vehicle-treated stroke animals. In other words, our results suggest that the inflammatory response in the brain was reduced in ProtheraCytes-treated stroke animals, but significant difference was only observed when ProtheraCytes was delivered intracerebrally. Immunohistochemical analyses of Iba-1 and CD34 in the peri-infarct area of ischemic brain revealed higher quantities (*F*_5,114_ = 274.7, ****P < .0001) of CD34 marker in transplantation cell group of stroke animals (Fig. 5). IC transplanted treatment group possessed the highest quantity of CD34 + cells, with IA transplanted treatment group the second highest, and IN transplanted treatment group had the lowest number of CD34 + cell count in the peri-infarct area.

### ProtheraCytes Promote the Formation of De Novo Neurons After Stroke

**Figure 6.**
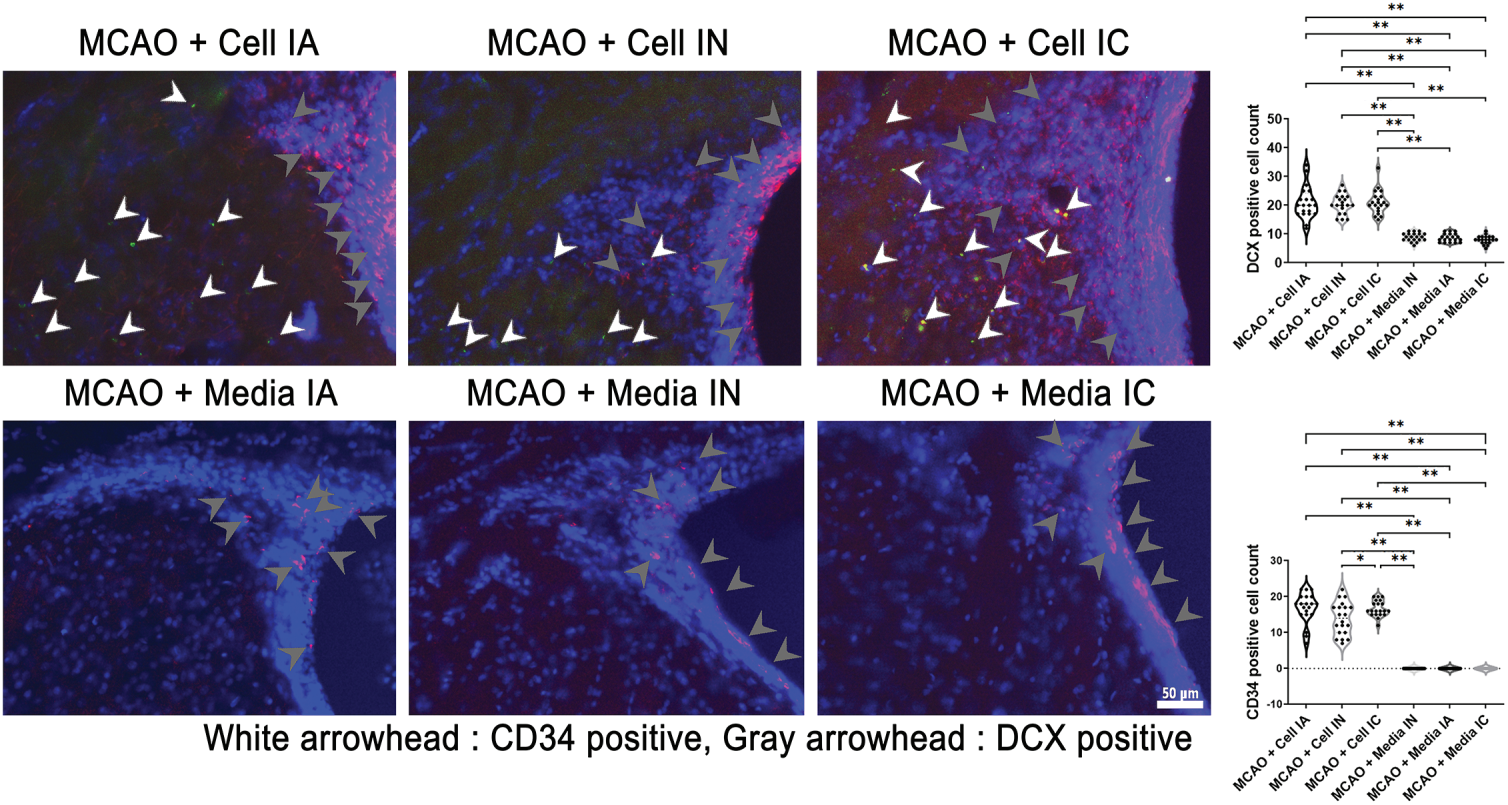
Neurogenesis. DCX double stained with CD34 measured neurogenesis differences between groups. All treatment groups MCAO + Cell IA, MCAO + Cell IN, and MCAO + Cell IC had significantly higher DCX expression than control groups MCAO + Media IA, MCAO + Media IN, and MCAO + Media IC (**P < .01). Treatment groups had significantly more CD34 expression (**P < .01). MCAO + Cell IN displayed significantly lower CD34 expression compared to MCAO + Cell IC (*P < .05). Scale bar = 50 µm.

DCX double stained with CD34 was used to assess neurogenesis in the peri-infarct area (Fig. 6). ProtheraCytes-treated stroke animals showed significant improvement of DCX positive cell count compared to vehicle-treated stroke animals (*F*_5,114_ = 81.65, ****P < .0001). Meanwhile, there was no significant difference in DCX positive cells within treatment group of different delivery routes. Altogether, the results indicate that significantly more de novo neurons were made in ProtheraCytes-treated stroke animals compared to vehicle-treated stroke animals, regardless of the method of transplantation. Immunohistochemical analyses of DCX and CD34 in the peri-infarct area of ischemic brain revealed larger number (*F*_5,114_ = 212.6, ****P < .0001) of CD34 marker in transplantation cell group of stroke animals compared to vehicle-treated groups (Fig. 6). IA transplanted treatment group displayed the highest quantity of CD34 + cells, while IC transplanted treatment group was the second highest, and IN transplanted treatment group had the lowest number of CD34 + cell count in the peri-infarct area.

### ProtheraCytes Induce Vasculogenesis in Stroke-Affected Brains

**Figure 7.**
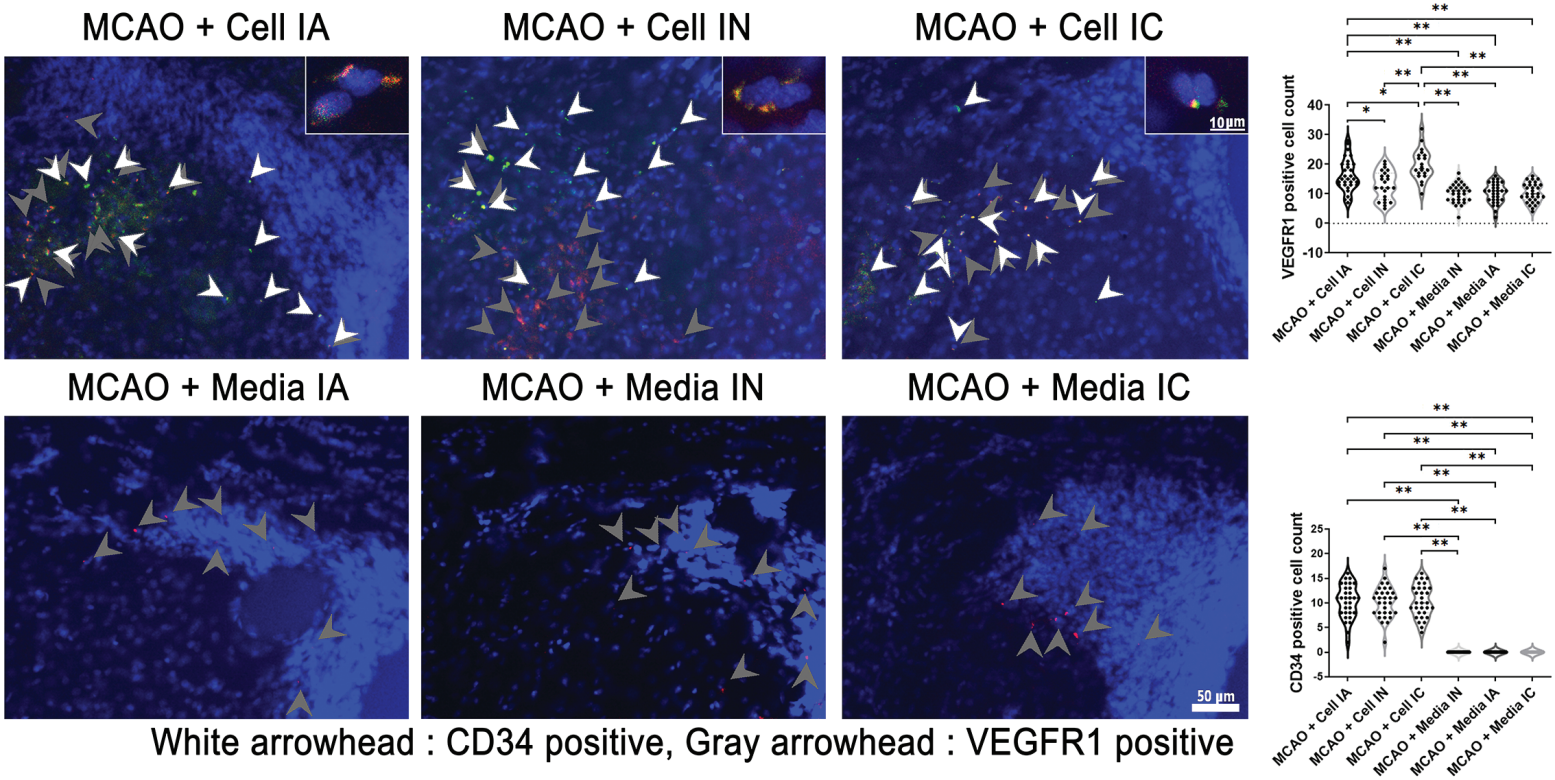
Angiogenesis. VEGFr1 double stained with CD34 quantified angiogenesis activity. MCAO + Cell IC and MCAO + Cell IA had significantly higher VEGFr1 expression than all MCAO + Media groups (**P < .01). MCAO + Cell IC had significantly more VEGFr1 positive cell counts than MCAO + Cell IA (*P < .05) and MCAO + Cell IN (**P < .01). MCAO + Cell IN had significantly less angiogenesis activity when compared to MCAO + Cell IA (*P < .05). CD34 expression was significantly higher in all treatment groups than in all media groups (**P < .01). Scale bar = 50 µm and 10 µm.

Angiogenesis in the peri-infarct area was assessed by marking VEGFr1 double stained with CD34 (Fig. 7). Stroke animals receiving ProtheraCytes through IA or IC methods showed significantly higher VEGFr1 positive cell counts compared to all vehicle-treated animal groups (*F*_5,161_ = 21.30, ****P < .0001). While stroke animals treated with IN-delivered ProtheraCytes had more VEGFr1 positive cells than vehicle-treated stroke animals, no significant difference was found. Additionally, within treatment groups, IC-delivered ProtheraCytes had significantly more VEGFr1 expression than in ProtheraCytes-treated stroke animals via IA (*P < .05) and IN (****P < .0001). Stroke animals administered with IA ProtheraCytes also had significantly higher VEGFr1 positive cell counts compared to stroke animals treated with IN ProtheraCytes. Our results display increased angiogenesis activity in ProtheraCytes-treated stroke animals compared to vehicle-treated stroke animals, where significant improvements were observed in animals transplanted with ProtheraCytes intracerebrally and intraarterially. Immunohistochemical analyses of VEGFr1 and CD34 in the peri-infarct area of ischemic brain showed positive stain in transplantation cell groups of stroke rats. The high magnification of staining showed CD34 neighboring VEGFr1 markers in positive cells.

### ProtheraCytes Possess Extracellular Cesicles

**Figure 8.**
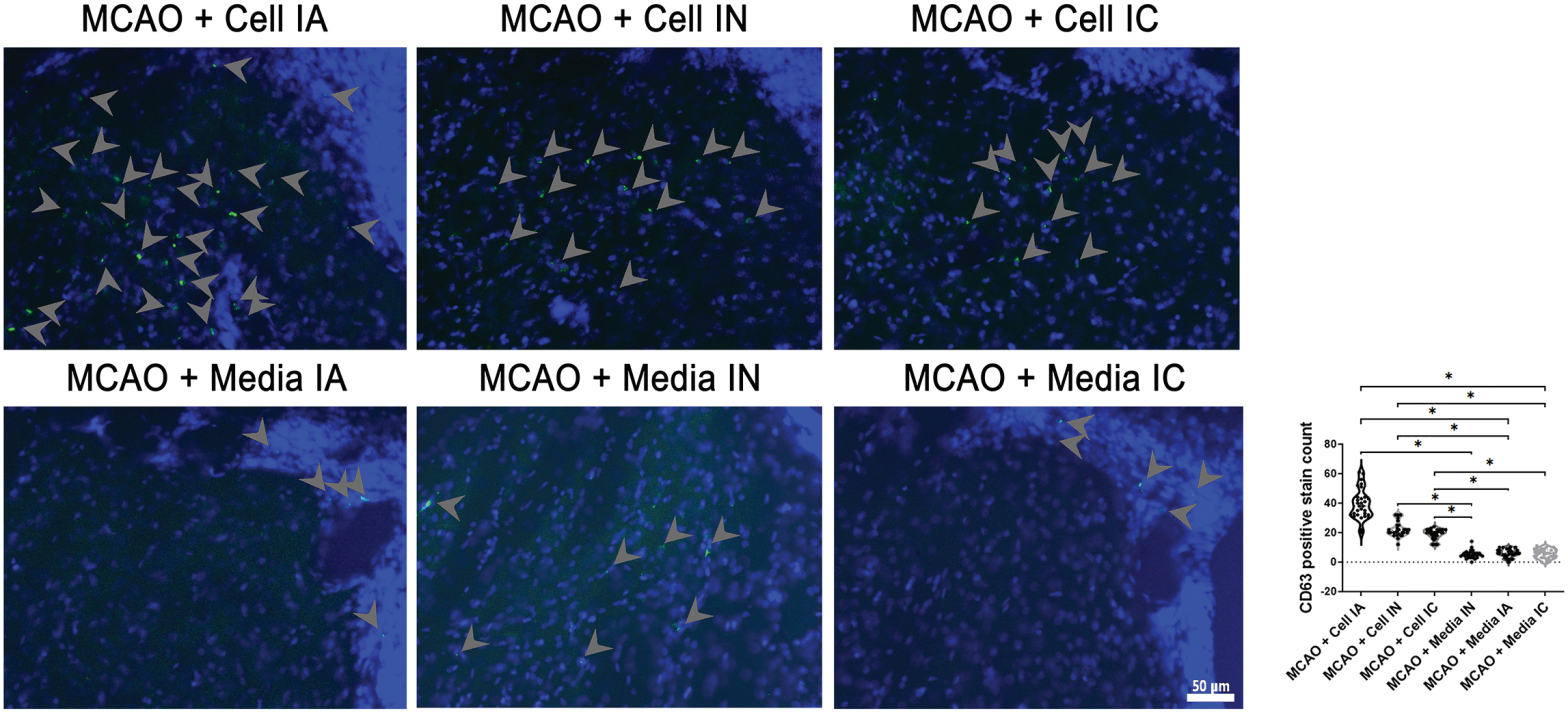
Extracellular vesicles. CD63 staining was used as a marker to quantify extracellular vesicles and healthy stem cell activity. All treatment groups MCAO + Cell IA, MCAO + Cell IN, and MCAO + Cell IC had significantly higher CD63 positive stain count than control groups MCAO + Media IA, MCAO + Media IN, and MCAO + Media IC (*p < 0.05). Scale bar = 50 µm.

**Figure 9.**
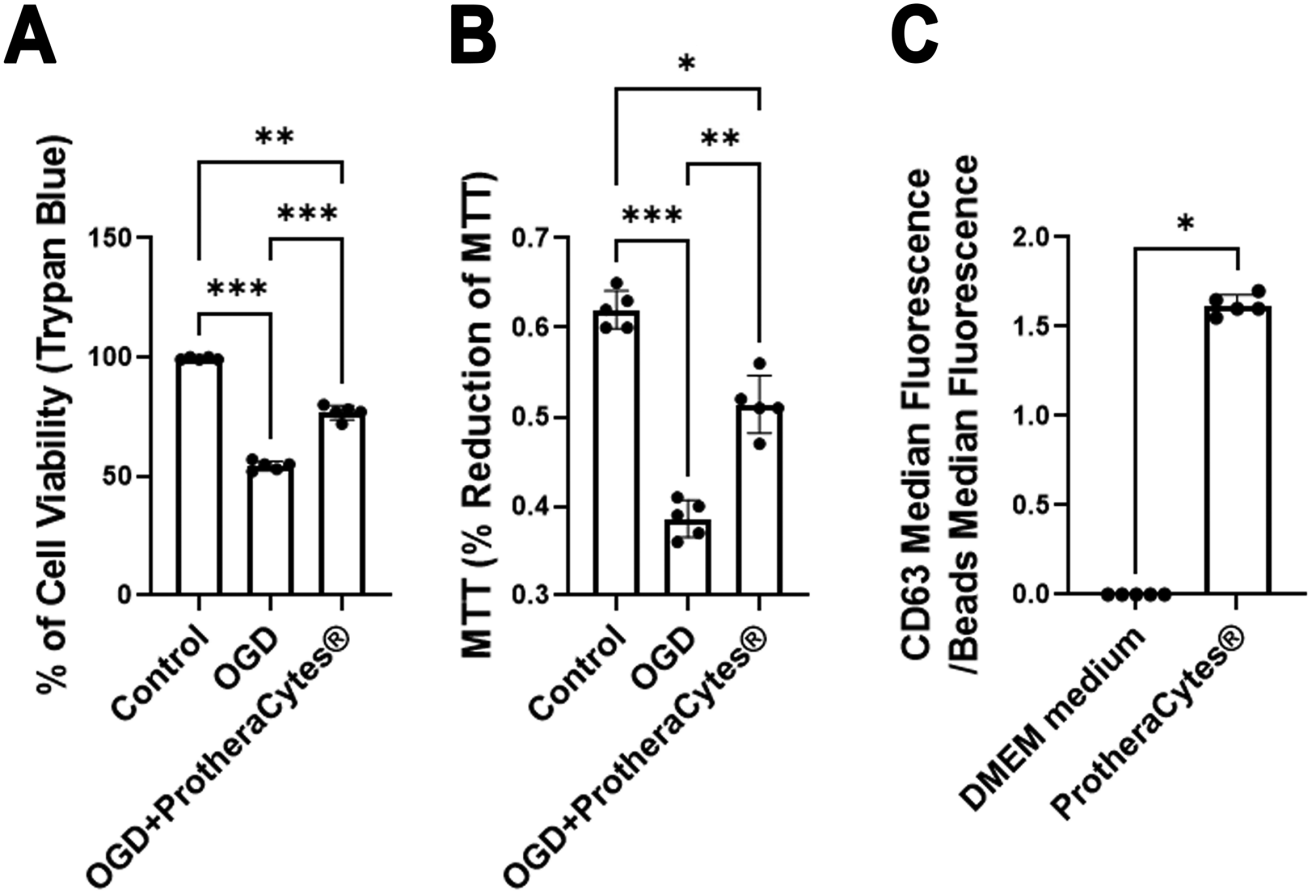
ProtheraCytes protect against in vitro stroke model of oxygen glucose deprivation. (**A**) Trypan blue. ANOVA revealed significant treatment effects. Post hoc Bonferonni’s tests revealed significant differences in cell survival, with primary neurons exposed to OGD and cocultured with ProtheraCytes rescuing against OGD-induced cell death significantly better than primary neurons subjected to OGD. (**B**) MTT. Similarly, ANOVA showed significant treatment effects, with post hoc Bonferonni’s tests detecting significant differences in metabolic activity, again with primary neurons exposed to OGD and cocultured with ProtheraCytes reducing the OGD-induced metabolic impairment significantly better than primary neurons subjected to OGD. (**C**) CD63. Analysis of exosome marker expression revealed that ProtheraCytes express the exosomal marker CD63, which was not detectable in the standard medium. Statistical significance is depicted as follows: *P < .05; **P < .01; ***P < .001.

Extracellular vesicles were assessed by utilizing CD63 markers (Fig. 8).^67^ Stroke animals treated with stem cells (IC, IN, and IA) showed significantly higher CD63 positive stain count compared to vehicle-treated stroke animals (*F*_5,174_ = 197.6, ****P < 0.0001). The results indicate that significantly more EVs and exosome activity were seen in ProtheraCytes-treated stroke animals compared to vehicle-treated stroke animals, regardless of the method of transplantation. Immunohistochemical analyses of CD63 revealed significantly higher quantities of healthy cells in the peri-infarct area. In parallel, under the in vitro stroke model of OGD, cocultures of ProtheraCytes with primary neurons protected against OGD as evidenced by increased neuronal cell viability and metabolic activity using trypan blue and MTT assays, respectively (Fig. 9A, 9B). Subsequent analysis of exosome marker expression revealed that ProtheraCytes expressed the exosomal marker CD63 (Fig. 9C), further supporting our postulated EV-mediated mechanism underlying the therapeutic effects of ProtheraCytes.

## Discussion

The present study investigated the potential therapeutic effects of ProtheraCytes through different transplantation paths, namely IC, IA, and IN. Transplanting ProtheraCytes significantly reduced behavioral and motor deficits in rats induced with transient MCAO when compared to the media groups, and there was no significant difference between treatment groups even after day 28. Behavioral improvements of ProtheraCytes-treated animals were reflected in Nissl staining of the ipsilateral striatum, where the infarct area was significantly reduced, and significantly higher percentage of live cells was seen in cell-treated groups compared to vehicle-treated groups. Despite similarities found in behavioral tests and Nissl staining, differences in immunohistochemistry were found between each transplantation method. Although neuroinflammatory marker Iba-1 was reduced in all MCAO + Cell groups, only IC transplantation significantly reduced Iba-1. Other reports have shown that CD34 + cells decreased sustained pro-inflammatory activity of NF-κB and its downstream effector molecules TNF-α, IL-1β, and IL-6 at the wound bed of diabetic NOD/SCID mice.^68^ Additionally, angiogenesis marker VEGFr1 expression were significantly higher in IC and IA cell-treated groups than vehicle-treated stroke animals but not IN. This suggests that IC and IA routes enhanced VEGF receptor phosphorylation, possibly by activating pro-angiogenesis signaling cascades in the ischemic brain injury.^69,70^ Angiogenesis could also be strengthened by direct cell-cell contacts leading to increased propagation of pro-angiogenetic stimuli.^42^ On the other hand, transplanting ProtheraCytes via IC, IA, and IN compared to all vehicle groups significantly enhanced DCX expression, a notable cytoskeleton associated protein that is transiently expressed during generation of neuronal cells. Furthermore, cell-treated groups, regardless of delivery route, had significantly higher CD63 marker quantities compared to vehicle-treated groups, indicating improved healthy cell activity. Despite varying results in the immunofluorescence analyses, IC, IA, and IN cell-treated stroke animals all displayed co-localization of CD34 with DCX and Iba-1, and CD34 was observed to be neighboring VEGFr1 markers in positive cells.

While transplanting stem cells 24 hours after MCAO is proposed as the most optimal time to treat stroke animals compared to earlier timeframes,^71^ broader time windows of stem cell therapy have been shown to have the same therapeutic effects in stroke models. Previously, we have shown significant reduction in infarct and peri-infarct areas, and decreased loss of hippocampal neurons upon treatment of stroke animals 60 days after the injury,^72^ suggesting a flexible timeframe of stem cell therapy and addressing the limited time window in current stroke treatments. Other studies demonstrated that IA delivery of mononuclear human umbilical cord blood cells (hUCB) significantly improved neurological performances in rats treated 24 hours or 7 days after stroke.^35^ Similar improvements in behavioral and physiological outcomes have been observed in IV administration at 48 hours after MCAO.^73^ To expand our earlier findings, our study treated animals with ProtheraCytes at 3 days after MCAO. In line with previous studies, stroke animals transplanted with stem cells at this delayed period displayed significant behavioral and neurological outcomes.

Currently, numerous routes of stem cell transplantation have been examined in MCAO models, but the most optimal method is yet to be determined. IC delivery of stem cells has been suggested as the most effective route as direct injection migrates cells to the infarct area and may improve neurological functions.^74^ Our present findings align with this claim as IC delivered ProtheraCytes -treated stroke animals had significant improvement in behavioral tests and surviving neurons, and significant reduction in the infarct area. Immunohistochemistry also revealed anti-inflammatory effects, which was significant in the IC delivered cell group, coupled with neurogenic and angiogenic properties in all transplanted groups. However, IC administration may not be suitable in clinical settings because of the invasiveness of the procedure and the intolerance of some stroke patients, which may lead to severe surgical side effects.^47,75-77^ Additionally, uncontrolled volumes of neural stem cells may cause adverse effects, further injuring healthy brain tissue.^78^ IV delivery was introduced as a less invasive method of stem cell transplantation, making it a safer clinical option compared to the IC route. Unfortunately, IV as well as IA administration sacrifices efficacy as stem cells typically migrate to organs other than the brain, such as the spleen, liver, lungs, heart, or kidney.^77,79,80^ Despite displaying endogenous regeneration in vesicles and neurons in IA transplanted cell groups, the IA route poses a risk of thrombus. Although studies have suggested that optimizing the stem cell and vehicle volumes and rate of injection, as in the present study, may limit the risk of microthrombus,^16,81-83^ IA routes still harbor a risk that limit its candidacy as a safe and effective stroke treatment method. IN administration is a relatively new administration route that has shown promising results. IN delivered bone marrow-derived MSCs were reported to pass through the blood-brain barrier and reach the lesion site in the brain without migrating to peripheral organs.^53^ Our results indicate therapeutic outcomes in motor functions and histopathology in IN-transplanted stroke-induced animals, suggesting IN as a potent delivery route. By overcoming major clinical hurdles in common delivery routes, IN transplantation may be the most practical method of transplanting stem cells, warranting further research should explore the potential use of the IN pathway.

Ongoing studies have revealed that ProtheraCytes secrete vascular endothelial growth factor (VEGF) and its concentration is significantly correlated with the number of CD34 + cells obtained after expansion (unpublished data). ProtheraCytes secrete exosomes with an average size of 86.7 ± 10.17 nm that express the exosomal markers CD9, CD63, and CD81. These exosomes contain proangiogenic miRNAs (126, 130a, 378, 26a), antiapoptotic miRNAs (21 and 146a), and antifibrotic miRNAs (133a, 24, 29b, 132). ProtheraCytes also exhibit in vitro angiogenic activity, express surface markers of endothelial progenitor cells, and can differentiate in vitro into endothelial cells.

The present study demonstrated that ProtheraCytes can be used as an effective stem cell treatment to reduce motor and histopathological deficits in ischemic stroke. Even through minimally invasive routes, such as IN transplantation, ProtheraCytes promote neuroprotective activities, including anti-inflammation, angiogenesis, neurogenesis, and extracellular vesicle activity. In the clinical setting, IN stem cell transplantation may be most suitable for treating ischemic stroke injuries especially in severely debilitated patients.

## Funding

This study was funded by CellProthera.

## Data Availability

All data reported in this study are available from the corresponding author on reasonable request.
